# Genetic and Toxigenic Variability within *Aspergillus flavus* Population Isolated from Maize in Two Diverse Environments in Kenya

**DOI:** 10.3389/fmicb.2018.00057

**Published:** 2018-01-26

**Authors:** Sheila Okoth, Marthe De Boevre, Arnau Vidal, José Diana Di Mavungu, Sofie Landschoot, Martina Kyallo, Joyce Njuguna, Jagger Harvey, Sarah De Saeger

**Affiliations:** ^1^School of Biological Sciences, University of Nairobi, Nairobi, Kenya; ^2^Department of Bioanalysis, Faculty of Pharmaceutical Sciences, Ghent University, Ghent, Belgium; ^3^Department of Applied Bioscience Engineering, Faculty of Bioscience Engineering, Ghent University, Ghent, Belgium; ^4^Biosciences Eastern and Central Africa, International Livestock Research Institute, Nairobi, Kenya; ^5^Feed the Future Innovation Lab, Kansas State University, Manhattan, KS, United States

**Keywords:** *Aspergillus flavus*, ITS, β-tubulin gene, calmodulin, aflatoxin biosynthesis genes, screening, metabolites, maize

## Abstract

*Aspergillus flavus* is the main producer of carcinogenic aflatoxins in agricultural commodities such as maize. This fungus occurs naturally on crops, and produces aflatoxins when environmental conditions are favorable. The aim of this study is to analyse the genetic variability among 109 *A. flavus* isolates previously recovered from maize sampled from a known aflatoxin-hotspot (Eastern region, Kenya) and the major maize-growing area in the Rift Valley (Kenya), and to determine their toxigenic potential. DNA analyses of internal transcribed spacer (ITS) regions of ribosomal DNA, partial β-tubulin gene (benA) and calmodulin gene (CaM) sequences were used. The strains were further analyzed for the presence of four aflatoxin-biosynthesis genes in relation to their capability to produce aflatoxins and other metabolites, targeting the regulatory gene aflR and the structural genes aflP, aflD, and aflQ. In addition, the metabolic profile of the fungal strains was unraveled using state-of-the-art LC-MS/MS instrumentation. The three gene-sequence data grouped the isolates into two major clades, *A. minisclerotigenes* and *A. flavus*. *A. minisclerotigenes* was most prevalent in Eastern Kenya, while *A. flavus* was common in both regions. *A. parasiticus* was represented by a single isolate collected from Rift Valley. Diversity existed within the *A. flavus* population, which formed several subclades. An inconsistency in identification of some isolates using the three markers was observed. The calmodulin gene sequences showed wider variation of polymorphisms. The aflatoxin production pattern was not consistent with the presence of aflatoxigenic genes, suggesting an inability of the primers to always detect the genes or presence of genetic mutations. Significant variation was observed in toxin profiles of the isolates. This is the first time that a profound metabolic profiling of *A. flavus* isolates was done in Kenya. Positive associations were evident for some metabolites, while for others no associations were found and for a few metabolite-pairs negative associations were seen. Additionally, the growth medium influenced the mycotoxin metabolite production. These results confirm the wide variation that exists among the group *A. flavus* and the need for more insight in clustering the group.

## Introduction

The filamentous fungus *Aspergillus flavus* is a cosmopolitan soil-born saprophytic organism with opportunistic parasitic behaviors to plants, animals and humans. *A. flavus* is the most common causal agent of aflatoxin (AF) contamination in food and feed, and the main producer of carcinogenic aflatoxin B1 (AFB1), (International Agency for Research on Cancer (IARC), 2002). *A. flavus* is known as the second cause of human invasive aspergillosis after *A. fumigatus* (Denning, 1988; Latge, 1999; Hedayati et al., 2007). Variability in the *A. flavus* phenotype exists, for example, in sclerotia formation, culture characteristics, and ability to produce aflatoxins. Reports illustrate that other metabolites can be produced with an unraveled toxicological profile (Pildain et al., 2008; Nicholson et al., 2009; Ehrlich and Brian, 2014). Culture and molecular methods have been used to group isolates into aflatoxigenic and non-aflatoxigenic strains (Zarrin and Erfaninejad, 2016), and to investigate the variability in fungal species and subspecies (Geiser et al., 1998a,b, 2000; Frisvad et al., 2007; Jurjević et al., 2015). Frisvad et al. (2005) raised *A. flavus* var. *parvisclerotigenus* to another species level, based on its atypical S-type sclerotia, profile of excreted metabolites and difference in rDNA-sequence. Geiser et al. (2000) subdivided *A. flavus* into Group 1 and Group 11 on the basis of sclerotia type and aflatoxin profile. Several authors have given evidence that *A. flavus sensu lato* may consist of several species (Geiser et al., 1998a,b, 2000; Pildain et al., 2008). The variation within the *A. flavus* isolates suggests the need for an extended taxonomic delineation for the benefit of the development of effective and safe biocontrol application technologies.

Among the mycotoxins produced by *A. flavus*, the production of aflatoxins have been studied more with the objective of understanding the diversity of the fungus, management of aflatoxin production by applying biocontrol technique and flow of genes involved in aflatoxin synthesis within a population. The aflatoxin biosynthetic pathway in *A. flavus* and A*. parasiticus*, involves approximately 30 genes clustered together in a 70-kb DNA region of the fungal genome on chromosome 111 roughly 80-kb away from telomere (Yu, 2012). The order of genes within the cluster is reported to be highly conserved wihin *Aspergillus* section Flavi. Among the 30 genes identified the functions of 19 have been assigned (Yu and Ehrlich, 2011). PCR detection of aflatoxin biosynthetic gene presence or expression has been used as diagnostic tool for aflatoxigenic fungi in selected commodities (Geisen, 2007). Sequence variability and deletions in various genes/regions of the aflatoxin biosynthetic cluster have also been used to determine the polyphyletic assemblages of *A. flavus* genotypes (Chang et al., 2005, 2006; Mohankumar et al., 2010; Gonçalves et al., 2012; Al-Wadai et al., 2013). Gene deletions and presence of single nucleotide polymorphisms (SNPs) in aflatoxin biosynthetic genes have been associated with *A. flavus* inability to produce aflatoxins. (Solorzano et al., 2014) observed genetic diversity among a population of *A. flavus* in a field in Mississipi over years and reported mutations among toxigenic isolates resulting into loss of ability to produce aflatoxins. Horizontal transfer of this gene cluster has also been documented (Susca et al., 2017). In this study the presence of the structural genes *aflD* (*nor-1*) which encodes the ketoreductase needed for the conversion of the 1'-keto group in norsolorinic acid (the first stable aflatoxin precursor) to the 1′-hydroxyl group of averantin; *aflP (omtA*) involved in the conversion of sterigmatocystin (ST) to *O*-methylsterigmatocystin (OMST) and demethylsterigmatocystin (DMST) to dihydro-*O*-methylsterigmatocystin (DHOMST); *aflQ* (*ordA*) involved in the conversion of OMST to AFB_1_ and AFG_1_ and of DHOMST to AFB_2_ and AFG_2_; and the regulatory gene (aflR) are determined in *A. flavus* strains collected from Eastern and Rift valley regions of Kenya.

Kenya has experienced several fatal acute aflatoxicosis outbreaks following consumption of contaminated food and feed (Okoth, 2016). Most of these outbreaks have occurred in the Eastern part of the country, after consumption of home-grown maize. This region is reported to harbor a large population of the S-strain of *A. flavus* (Okoth et al., 2012). This study sought to establish the variability patterns of *A. flavus* strains previously isolated from the Eastern and Rift Valley regions of Kenya using sequence analysis of the ITS region, and parts of the β-tubulin and calmodulin genes and metabolite profiles (Okoth et al., 2012). The Rift Valley is the major commercial maize-growing area of Kenya, and no aflatoxicosis outbreaks have been reported there. Such information is important given that aflatoxin production is a polygenic variable trait that may experience outcrossing between two strains during reproduction. Reproduction in *A. flavus* is mostly asexual, however sexual reproduction is also known to occur together with non-sexual recombination among isolates that are highly similar. The mechanisms are not clear, and can only be deduced by population studies to unravel the diversity of the fungus. This is the first comprehensive analysis of *A. flavus* isolates in Kenya using molecular analysis and metabolic profiling. The possible variations within *A. flavus* isolates is explained here using the sequence analysis of the conserved ITS region of the rDNA gene cluster and parts of the calmodium and β-tubulin genes, presence of four genes in the aflatoxin biosynthetic pathway and metabolite profile.

## Materials and methods

### Source of *Aspergillus flavus* isolates

The isolates used in this study were previously recovered from maize collected from farmers in the Rift valley (*n* = 255) and the Eastern regions of Kenya (*n* = 258) in 2010, from a larger study carried out by Okoth et al. (2012) and Nyongesa et al. (2015). The isolates were identified as *A. flavus* using morphological and cultural characteristics. The isolates were preserved on silica gel at the University of Nairobi. From this collection, 109 isolates [*n* = 53 (Eastern Kenya), *n* = 56 (Rift Valley)] were randomly selected, and grown at 28°C on potato dextrose agar (PDA).

### Multi-analysis of *Aspergillus* metabolites

#### Reagents and chemicals

The individual mycotoxin solid calibration standards (1 mg) of aflatoxin B1(AFB1), aflatoxin B2 (AFB2), aflatoxin G1 (AFG1), aflatoxin G2 (AFG2), and zearalanone (ZAN) (internal standard) were obtained from Sigma Aldrich (Bornem, Belgium) Zearalanone was used as internal standard to quantify the AFs, but also to feature as identification criterium for the other metabolites. One of the identification criterium is the relative retention time. The retention time of the internal standard is compared to the retention time of the metabolites, and should remain constant.

All mycotoxin solid standards were dissolved in methanol (1 mg/mL) and were storable for a minimum of 1 year at −18°C (Spanjer et al., 2008). The working solutions of AFB1, AFB2, AFG1, AFG2, and ZAN (10 ng/μl) were prepared in methanol, and stored at −18°C and renewed monthly. Water was obtained from a Milli-Q® SP Reagent water system from Millipore Corp. (Brussels, Belgium). Disinfectol® (denaturated ethanol with 5% ether) was supplied by Chem-Lab (Zedelgem, Belgium). Methanol (LC-MS grade) was purchased from BioSolve (Valkenswaard, the Netherlands), while acetonitrile (Analar Normapur) and ammonium acetate were obtained from VWR International (Zaventem, Belgium). Acetic acid (glacial, 100%) was supplied by Merck (Darmstadt, Germany).

#### Sample preparation, treatment, and extraction

*Aspergillus flavus* isolates analyzed in this study were grown on Czapek yeast extract agar (CYA) and aflatoxin-inducing yeast extract sucrose agar (YESA) to determine their metabolites profile. A loop of mycelia and spores of the isolates scraped from 9 mm PDA plugs were inoculated on 9 cm diameter Petri plates, and incubated at 28°C for 7 days in the dark. Using a 9 mm diameter sterile cork borer, 9 plugs were harvested uniformly from the plates into 50 mL propylene tubes. The agar was submitted to acetonitrile maceration (5 mL) overnight (15 h). Then, metabolites were extracted from the matrix with 10 mL of water and 20 mL of dichloromethane while shaking for 30 min. The mixture was centrifuged (3,000 g, 15 min), 10 mL from the bottom face was evaporated and resuspended with 1 mL of methanol/acetonitrile/water (30/30/40, v/v/v).

#### LC-MS/MS methodology

An LC-MS/MS method was developed and validated for the analysis of 32 analytes (Table 1). A Waters Acquity UPLC system coupled to a Xevo TQS mass spectrometer (Waters, Milford, MA, USA) was used to analyse the samples, equipped with MassLynx® (version 4.1) and QuanLynx® (version 4.1) software (Waters, Manchester, UK) for data acquisition and processing. A ZORBAX Eclipse XDB C18-column (1.8 μm, 100 × 2.1 mm) was applied (Agilent Technologies, Diegem, Belgium). The mobile phase consisted of water/methanol (95/5, v/v (A)) and methanol/water (95/5, v/v (B)), both adjusted with 5 mM ammonium acetate and 0.1% formic acid, at a flow rate of 0.4 mL/min. The gradient elution programme started at 100% mobile phase A for 0.5 min. Then, the mobile phase B increased with a linear increase to 99% in the 20 min. The mobile phase B was kept at 99% for 5 min. The mobile phase linearly decreased till 0% for 1 min. Mobile phase A (100%) was running for 4 min. The duration of each LC run was 30 min, including re-equilibration. The capillary voltage was 3 kV, and nitrogen was applied as spray gas. Source and desolvation temperatures were set at 120° and 400°C, respectively. The argon collision gas pressure was 9 × 10^−6^ bar, the cone gas flow 35 L/h and the desolvation gas flow 800 L/h. For increased sensitivity and selectivity, the instrument was operated in the selected reaction monitoring (SRM) mode, and two SRM transitions were monitored for each analyte. The SRM-transitions for every analyte are described in Table 1. Matrix-matched calibration plots were constructed for the determination of the AFB1, AFB2, AFG1, and AFG2. ZAN was used as internal standard in the multi-mycotoxin analysis. The limit of detection (LOD) was calculated as three times the standard error of the intercept, divided by the slope of the standard curve; the limit of quantification (LOQ) was computed in a similar way, except for the standard error, which was by a factor of six. The calculated LOD and LOQ were verified by the signal-to-noise ratio (s/n), which was more than 3 and 10, respectively, in accordance with the IUPAC guidelines (IUPAC, 1995).

**Table 1:** Optimized ESI-MS/MS parameters for the confirmation and quantification of analyzed metabolites.

**Metabolite**	**Precursor ion (m/z)**	**Product ions^a^ (m/z)**	**CE^a^^,^^b^ (eV)**	**CV^c^ (V)**	**Retention time (min)**	**LOD^d^ (μg/kg)**	**LOQ^e^ (μg/kg)**
Kojic acid	143.0	69.1/125.3	30/30	35	4.1	n.a.^f^	n.a.
Flavacol	209.2	95.1/123.0/137.1	40/35	35	13.5	n.a.	n.a.
Aspergillic acid	225.2	165.1/207.2	40/35	35	12.5	n.a.	n.a.
Hydroxyneoaspergillic acid	241.2	137.1/163.0/191.1	40/35	35	13.2	n.a.	n.a.
m-isocoumarin	307.1	149.1/27.1	35/30	35	10.4	n.a.	n.a.
Aflatoxin B1	313.1	270.9/285.1	35/22	70	11.6	0.016	0.049
Aflatoxin B2	315.1	286.9/	35/40	25	10.9	0.017	0.049
STE analog	325.1	281.5/310.1	34/24	35	11.3	n.a.	n.a.
STE	355.1	281.1/310.1	34/24	35	15.6	n.a.	n.a.
Aflatoxin G1	329.1	243.5/311.3	25/20	35	10.1	0.010	0.033
Aflatoxin G2	331.1	245.1/313.1	18/19	25	9.5	0.090	0.032
Leporin C	336.2	200.1/214.1	40/40	35	18.3	n.a.	n.a.
CPA	337.2	140.1/196.1	40/35	35	17.2	n.a.	n.a.
OMST	339.1	306./324.1	35/30	35	14.5	n.a.	n.a.
β-CPA	339.2	154.1/198.1	40/40	35	16.4	n.a.	n.a.
DHOMST	341.1	285.1/326.1	35/30	35	13.5	n.a.	n.a.
Aspertoxin	355.0	322.1/340.1	30/35	35	12.3	n.a.	n.a.
Noranthrone	357.2	245.1/273.0	40/35	35	14.2	n.a.	n.a.
Versiconol	361.1	285.1/325.1	35/30	35	11.5	n.a.	n.a.
Speradine A	367.2	160.1/266.1	35/30	35	13.2	n.a.	n.a.
OxyHOMST	371.1	282.1/314.1	35/30	35	11.7	n.a.	n.a.
DHoxyHOMST	373.1	322.1/355.1	35/30	35	11.3	n.a.	n.a.
Versiconol Hem Acid	401.0	283.1/307.1	35/30	35	11.4	n.a.	n.a.
Aflavinine	406.3	180.2/388.3	35/30	35	13.2	n.a.	n.a.
Paspaline	422.3	158.1/386.4	45/26	35	20.3	n.a.	n.a.
Aflavarin analog 2	425.1	334.1/383.1	35/40	35	17.0	n.a.	n.a.
Di-OH Aflavinine	438.3	285.1/402.3	35/35	35	13.4	n.a.	n.a.
Aflavarin analog 1	439.1	361.1/397.1	40/35	35	13.2	n.a.	n.a.
Aflavarin	455	379.2/413.2	35/35	35	14.5	n.a.	n.a.
Paspalinine	434	130.1/376.1	40/35	35	16.0	n.a.	n.a.
Aflatrem	502.3	156.1/198.1	40/26	35	16.4	n.a.	n.a.
Ditryptophenaline	693.3	318.1/346.2	40/35	35	17.2	n.a.	n.a.
Zearalanone	321.0	189.0/303.0	22/14	35	7.59	n.a.	n.a.

a Values are given in order: quantifier ion/qualifier ion;

b CE, Collision energy;

c CV, Cone Voltage;

d LOD, Limit of Detection;

e LOQ, Limit of Quantification;

f *n.a., not applicable. M-isocoumarin, Methylcitro-isocoumarin; STE analog, Sterigmatocystin analog; STE, Sterigmatocystin; CPA, Cyclopiazonic acid; OMST, O-methylsterigmatocystin; β-CPA, β-Cyclopiazonic acid; DHOMST, Dihydro-O-methylsterigmatocystin; OxyHOMST, Oxy Dihydro-O-methylsterigmatocystin; DHoxyHOMST, Deoxy Dihydro-O-methylsterigmatocystin; Versiconol Hem Acid, Versiconal Hemiacetal Acetate*.

### Genotypic analysis

#### DNA extraction

Conidia of the *A. flavus* isolates grown on PDA for 5 days were inoculated into 250 mL Erlenmeyer flasks containing 100 mL of Wickerham medium (40 g/L glucose, 5 g/L peptone, 3 g/L yeast extract, 3 g/L malt extract). Incubation was carried out at 25°C under shaking conditions (150 rpm) for 2 days. Then, mycelia were harvested by filtration and washed with 0.5 M ethylenediamine tetra-acetic acid (EDTA) & sterile distilled water (dH_2_O), and freeze-dried at -70°C for DNA extraction. The mycelia were ground into a fine powder using a pestle and mortar. The powder (~100 mg) was transferred into a 2 mL sterile eppendorf tube containing 2 mm diameter glass beads. Then, 650 μL of lysis CTAB (cetyltrimethylammonium bromide) buffer was added and the mixture was ground in a tissue miller at a frequency of 30/s for 3 min after which the samples were incubated at 65°C for 1 h. Proteins were precipitated by adding 600 μL phenol, inverted gently and centrifuged at 14000 rpm for 20 min. The top aqueous layer was pipetted into a new tube. Afterwards, 600 μL phenol/chloroform (25/24, v/v) was added, inverted gently and centrifuged at 14000 rpm for 20 min. This procedure was repeated twice with 600 μL of chloroform. The clean supernatant was transferred to a new 1.5 mL eppendorf tube to which 60 μL 3M sodium acetate (pH 8) & 800 μL isopropanol were added, and incubated overnight at 4°C for precipitation. The samples were centrifuged at 14000 rpm for 10 min at 4°C. The supernatant was discarded, after which the DNA pellet was washed twice with 1 mL 70% ethanol, and centrifuged again at 14000 rpm for 5 min at 4°C. The DNA pellet was dried in an oven with the eppendorf tube lid open at 55°C for 30 min. The pellet was re-suspended in 80 μL low salt TE buffer, and 5 μL of RNase (10 mg/mL) was added to remove any RNA contamination. Depending on the yield 1 μL on the 1% agarose gel was transferred, and stored at −20°C. 50–100 ng of DNA was checked for intactness on 1% agarose. The ratio 260/280 = 1.6–1.8 and 260/230 = 1.8–2.2 obtained showed that the DNA was of food quality, and this was stored at −20°C.

#### PCR amplification and sequencing

PCR analysis of the internal transcribed spacers (ITS1–5.8S-ITS2 cluster) regions of the ribosomal DNA gene cluster, and parts of the B-tubulin and calmodulin genes was done for each isolate using the primer pairs shown in Table 2. PCR amplifications were performed on 25 μL of a reaction mixture containing MgCl_2_-free reaction buffer, 50 mM MgCl_2_, 10 mM dNTP mix, 10 μM of each primer, 5 U/μL Taq DNA polymerase and 5 ng/μL of template DNA. The AccuPower® Taq PCR PreMix (Bioneer, Korea) was preferred as the Taq in the premix is not a high fidelity enzyme.

**Table 2:** Sequences of the nucleotide primers used in the study.

**Primer code**	**Target gene**	**Primer sequences**	**PCR product Size (bp)**	**References**
ITS1F	ITS1–5.8S-ITS2 cluster	(5′-CTTGGTCATTTAGAGGAAGTAA-3′)	595	Jurjević et al., 2015
ITS4R		(5′-TCCTCCGCTTATTGATATGC-3′)		
Bt2	Partial β-tubulin gene (*ben*A)	Bt2a (5′-GGTAACCAAATCGGTGCTGCTTTC-3′)	550	Glass and Donaldson, 1995
Bt2b		Bt2b (5′-ACCCTCAGTGTAGTGACCCTTGGC-3′)		
CF1	Partial calmodulin gene (Ca*M*)	CF1M (5-AGGCCGAYTCTYTGACYGA)	700	Tam et al., 2014
CF4		CF4 (5′-TTTYTGCATCATRAGYTGGAC-3′)		
AflD-1for	aflD (nor-1)	Nor1-F (5′-ACC GCT ACG CCG GCA CTC TCG GCA C-3′)	862	Rodrigues et al., 2009
AflD-2rev		Nor1-R (5′-GTT GGC CGC CAG CTT CGA CAC TCC G−3′)		
AflQ-1for	aflQ (ordA)	Ord1-gF (5′-TTA AGG CAG CGG AAT ACA AG-3′)	757	Sweeney et al., 2000
AflQ-1for		Ord1-gR (5′-GAC GCC CAA AGC CGA ACA CAA A-3′)		
AflP-1for	aflP (omtA)	AflP-1for aflP (omtA) 5′-AGCCCCGAAGACCATAAAC-3′	870	Fakruddin et al., 2015
AflP-2rev		AflP-2rev 5′-CCGAATGTCATGCTCCATC-3′		
AflR-1for	aflR	AflR-1for aflR 5′-AAGCTCCGGGATAGCTGTA-3′	1,079	Fakruddin et al., 2015
AflR-2rev		AflR-2rev 5′-AGGCCACTAAACCCGAGTA-3′		

PCR was carried out as follows: (1) one step at 94°C for 3 min; (2) 30 cycles of the following three steps: 1 min at 94°C, 1 min at 57°C, 1 min at 72°C, and (3) one final 10-min step at 72°C. The PCR products were separated by 1.2% agarose gel electrophoresis in a Tris-base, acetic acid and EDTA buffer, and stained with ethidium bromide. PCR product purification was done using QIAquick PCR Purification Kit (Qiagen) and sequencing performed at Inqaba Ltd (Cape Town, Republic of South Africa). The obtained DNA sequences were assembled and edited using CLC Main Workbench 7.7.3 (https://www.qiagenbioinformatics.com/), and then compared with the sequences that are deposited in the GenBank (http://www.ncbi.nlm.nih.gov/). The sequences of each of the three genes were aligned separately with references in the NCBI database, and the SNPs/Indels were observed. Phylogenetic trees were constructed using maximum likelihood with 1000 bootstraps replicates using MEGA v 6. Sequences were deposited at GenBank under accession numbers indicated on Supplementary Tables 1–3.

#### Detection of aflatoxin genes

All *A. flavus* isolates were tested for the presence of structural genes aflD, aflQ, aflP, and regulatory genes aflR, using some of the primers described by Gallo et al. (2012), listed in Table 2, and optimized in the laboratory conditions. The aflD gene encodes an enzyme that catalyzes the conversion of the first stable aflatoxin biosynthesis intermediate, norsolorinic acid to averantin in both *A. flavus* and *A. parasiticus* (Bennett, 1981; Papa, 1982). Both aflP and aflQ are involved in the late steps of the AF pathway. However, aflP is involved in the conversion of sterigmatocystin to O-methylsterigmatocystin (omst) and dihydro-sterigmatocystin (dhst) to dihydro-O-methylsterigmatocystin (dhomst). The aflQ gene is involved in the conversion of O-methylsterigmatocystin (omst) to AFB1 & AFG1, and dihydro-O-methylsterigmatocystin (dhomst) to AFB2 & AFG2 in both *A. parasiticus* (Cleveland, 1989) and in *A. flavus* (Yu et al., 1998).

### Data analysis

For statistical evaluation, the R software package (R core Team, 2014) version 2.15.3 was used. To cluster the isolates based on their toxin profile and to cluster the toxins, a hierarchical clustering, based on the simple matching distance, which is a measure for the dissimilarity between binary sample sets, was used. The data are represented as heatmaps, where to color codes indicate the presence (green) or absence of a certain metabolite or toxin. Furthermore, based on the expected and observed co-occurrence of the toxin, pairwise associations were classified as negative, positive or random. To verify whether the clustering of the isolates based on the toxin profile on CYA and YES was similar, correlation analysis was performed applying the Mantel test.

## Results

### Multi-analysis of *Aspergillus* metabolites

**Figure 1:**
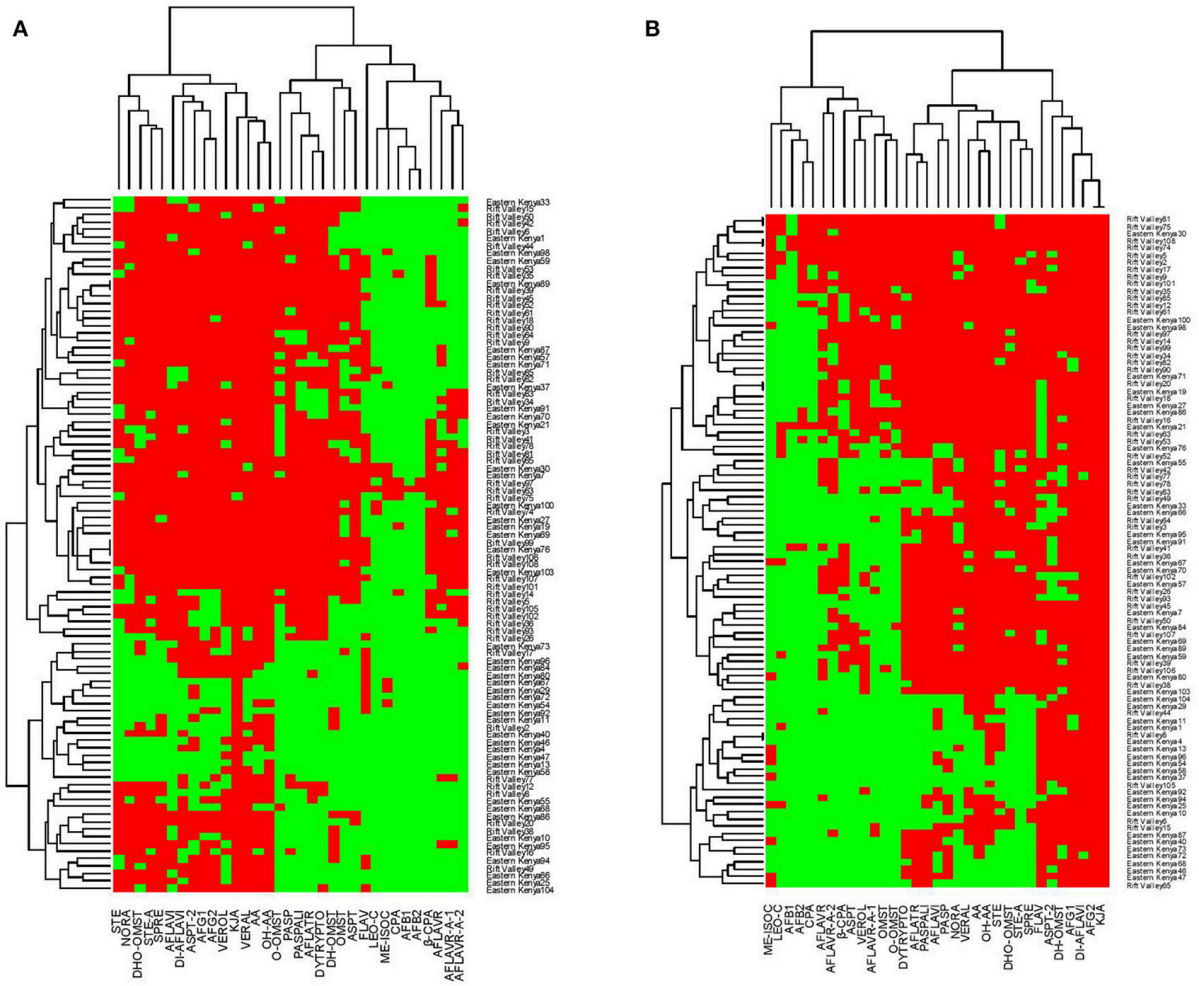
Hierarchical clustering of the isolates and toxins grown on either CYA **(A)** or YESA **(B)** medium.

**Figure 2:**
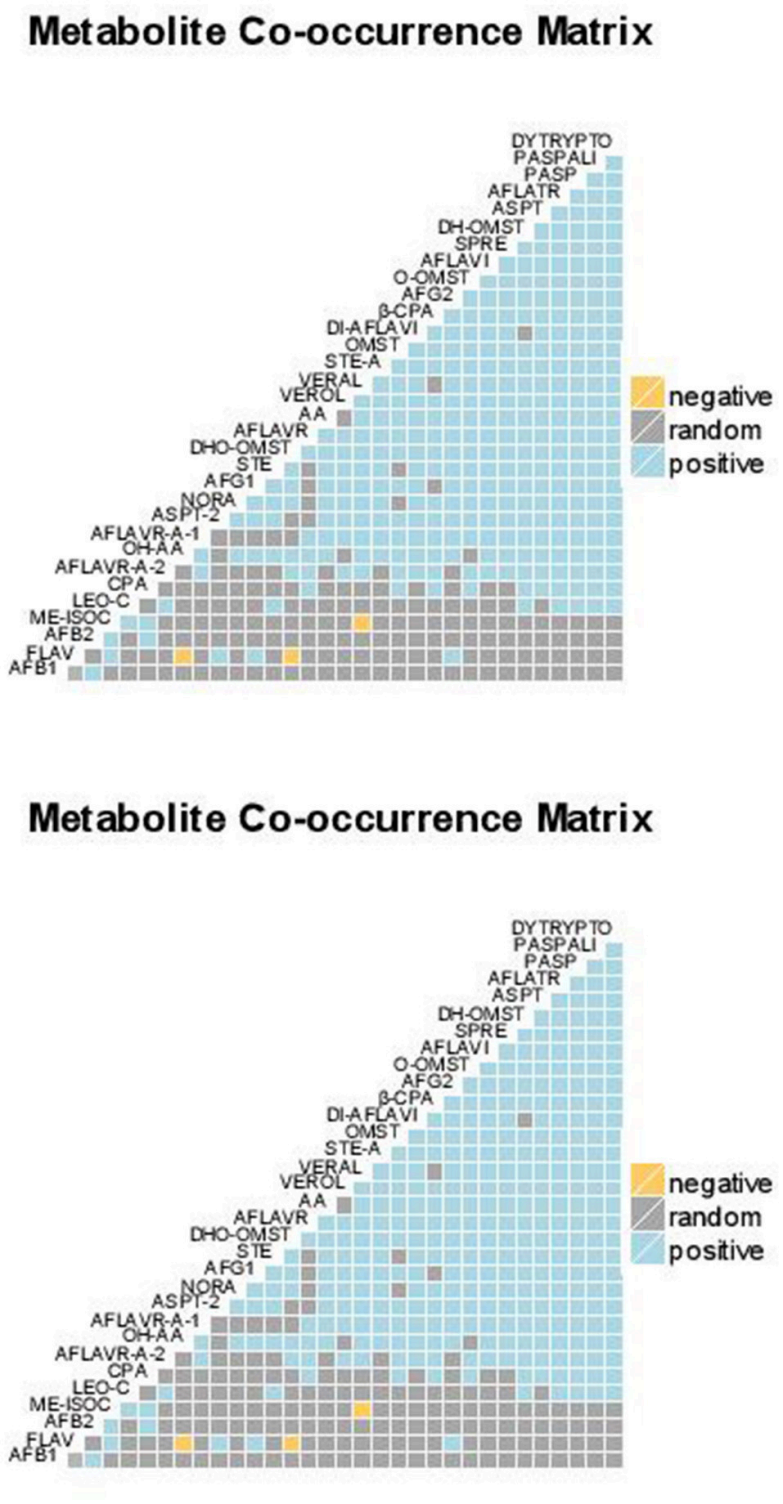
Co-occurrence of the observed metabolites (**A** CYA and **B** YESA). A positive association means that in case one metabolite is observed it is very likely that also the other metabolite will be present. A negative association means that in case one metabolite is observed it is very unlikely that also the other metabolite will occur. A random association means that nothing can be said concerning the co-occurrence of both metabolites.

To cluster the isolates based on their toxin profile and to cluster the toxins, a hierarchical clustering, based on the simple matching distance, which is a measure for the dissimilarity between binary sample sets, was used. The data are represented as heatmaps, where color codes indicate the presence (green) or absence of a certain metabolite or toxin. Furthermore, based on the expected and observed co-occurrence of the toxin, pairwise associations were classified as negative, positive or random. In Figure 1 the row dendrogram shows the clustering of the different isolates grown on either CYA or YESA medium. Isolates with a similar toxin profile were grouped together in the dendrogam. The dendrogram shows the associations between metabolites, metabolites with a similar occurrence pattern for the different isolates cluster together. To verify whether there was a correlation between the distances between isolates based on their toxin profile produced on CYA and the distance between isolates based on their toxin profile on YESA, a Mantel test with 1000 permutations was used (Figure 2). This test checks for significant correlations between distance matrices by a permutation procedure. The mantel correlation was 0.31, which was significant at α = 0.05. This means that the clustering of the isolates based on their toxin profile on CYA is correlated with the clustering of the species based on their toxin profile on YESA.

High positive correlations (*r* > 0.5) were observed between the following mycotoxin-pairs on the CYA-medium: AFB1-AFB2 (0.87); AFB1-STE (0.53); AFB1-CPA (0.67); AFB2-CPA (0.5); AFB1- OxyHOMST (0.69); AFB2- OxyHOMST (0.52); AFB1-β-CPA (0.59); AFB2-β-CPA (0.55); AFB1-aspertoxin (0.66); AFB2-aspertoxin (0.65); AFB1-noranthrone (0.88); AFB2-noranthrone (0.91); AFB1-versiconol (0.86) and AFB2-noranthrone (0.92). For the YESA-medium following positive correlations could be quantified: AFB1-AFB2 (0.88); STE-AFG1 (0.77); AFB1- OxyHOMST (0.52); AFB1-versiconol (0.52); AFB1-noranthrone (0.58) and AFB2- DHOMST (0.77). All the 109 isolates produced both AFB1 and AFB2 on CYA except genotypes 63 (did not produced both toxins) and 97 (produced only AFB1). On YESA, isolates 21, 41, 74, 108, and 109 did not produce aflatoxins while 13 isolates produced only AFB1. The remaining 91 isolates produced both AFB1 and AFB2. For the other metabolites, a great variability was observed in the profiles of the isolates represented by the many subclades in Figure 1.

### DNA analysis of *A. flavus* isolates

#### Identification and analysis of polymorphism in *A. flavus* b-tubulin gene

**Figure 3:**
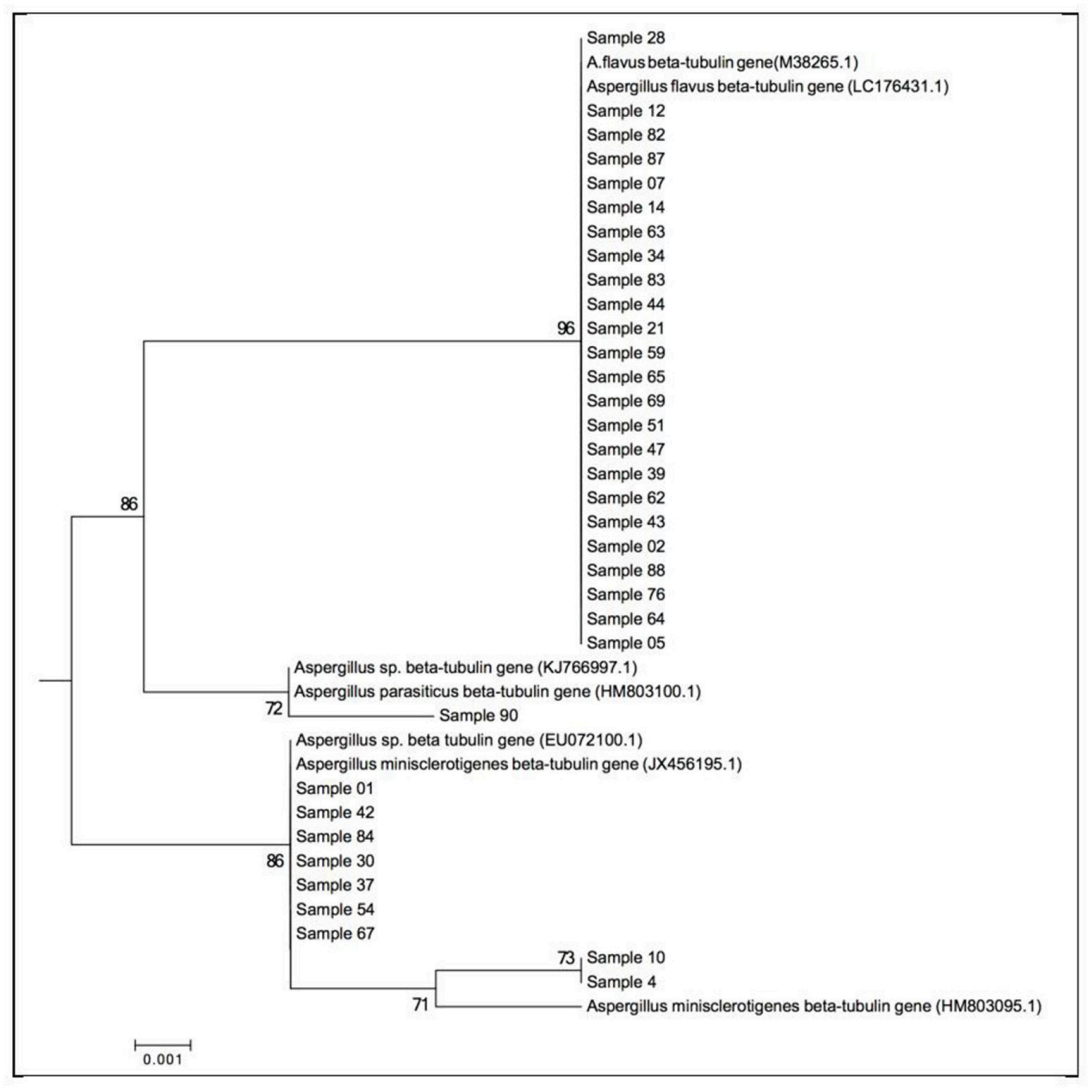
Molecular Phylogenetic analysis of the β-tubulin gene by the Maximum Likelihood method based on the Juke Cantors model (Nei and Kumar, 2000) at 1000 bootstraps. The tree is drawn to scale, with branch lengths measured in the number of substitutions per site. Representative isolates are used. Evolutionary analyses were conducted in MEGA6 (Tamura et al., 2013).

During analysis of the β-tubulin gene, 386 characters were analyzed among which 11 sites were found to be informative with confirmed polymorphism. Isolate 28 and 67 showed heterozygosity at nucleotide position 218 and 344, respectively. Single nucleotide polymorphism (SNP) and indels were observed as shown in Table 3. Most isolates were 100% identical to *A. flavus* sequences with an exception of isolates 1, 4, 10, 30, 37, 42, 54, 67, and 84, which have 100% identity to *A. minisclerotigenes*. All these isolates are from Eastern region of Kenya, except for isolate 42 which is non-sclerotia-forming (Rift Valley). The Eastern Kenya isolates are S-strain except for 84, which did not produce any sclerotia. Isolate 90 was 99% identical to *A. parasiticus* sequences deposited in the NCBI database. The neighbor joining (NJ) tree based on β-tubulin genes sequences indicates that *A. flavus* with *A. parasiticus* are closely related forming a clade distinct from *A. minisclerotigenes* (Figure 3).

**Table 3:** Nucleotide deletions and insertions in b-tubulin gene sequences from isolates of *Aspergillus* sp.

**Nucleotide position**	**Base substitution**	**Base insertion**	**Isolate**	**Identity - NCBI database**	**Identical NCBI accession numbers**
20	G with A		1, 4, 10, 30, 37, 42, 54, 67, 84	*A. minisclerotigenes* (100%)	JX456195.1 HM803083.1
			90	*A. parasiticus* (99%)	KX400742.1
34	A with G		1, 4, 10, 30, 37, 42, 54, 67, 84	*A. minisclerotigenes*	
			90	*A. parasiticus*	
52	G with A		90	*A. parasiticus*	
141	G with A		1, 4, 10, 30, 37, 42, 54, 67, 84	*A. minisclerotigenes*	
194	C with T		90	*A. parasiticus*	
218	C with C/T		28	*A. flavus* (100%)	KX400740.1
248	A with T		4, 10	*A. minisclerotigenes*	
249		A	4, 10	*A. minisclerotigenes*	
249		T	1, 30, 37, 42, 54, 67, 84	*A. minisclerotigenes*	
			90	*A. parasiticus*	
249	Indel		2, 5, 7, 12, 14, 2128, 34, 39, 43, 44, 47, 51, 59, 62, 63, 64, 65, 69, 76, 82, 83, 87, 88	*A. flavus* (100%)	KX400740.1
268	C with G		1, 4, 10, 30, 37, 42, 54, 67, 84	*A. minisclerotigenes*	
335	T with C		1, 4, 10, 30, 37, 42, 54, 67, 84	*A. minisclerotigenes*	
			90	*A. parasiticus*	
344	G with A/G		67	*A. minisclerotigenes*	
344	G with A		42	*A. minisclerotigenes*	

#### Identification and analysis of polymorphism in *A. flavus* calmodulin gene

**Figure 4:**
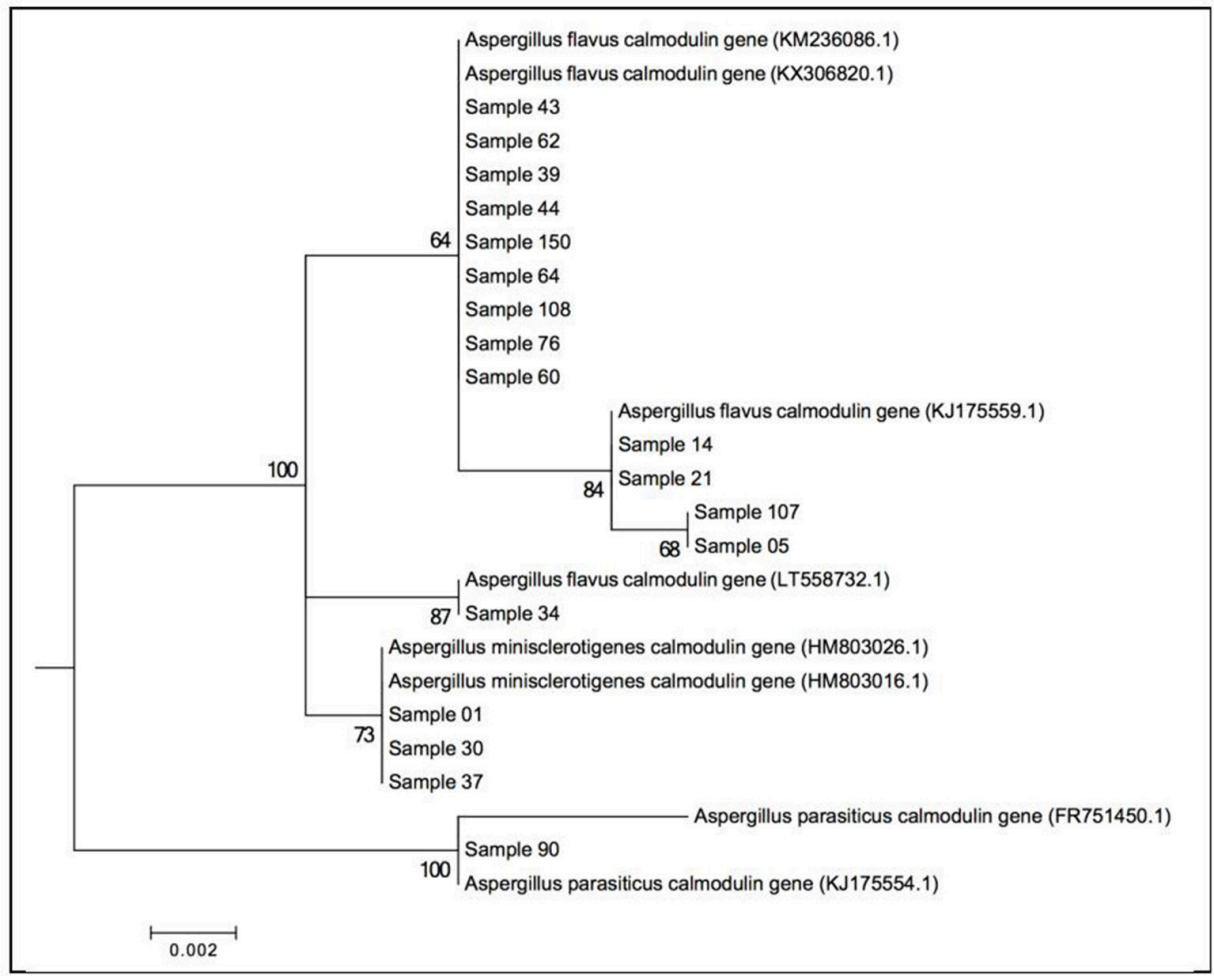
Molecular Phylogenetic analysis of the calmodulin gene by Maximum Likelihood method based on the Kimura 2-parameter model at (Jukes and Cantor, 1969) 1000 bootstraps. The tree is drawn to scale, with branch lengths measured in the number of substitutions per site. Representative isolates are used. Evolutionary analyses were conducted in MEGA6.

During the analysis of calmodulin gene sequences, 563 characters were used, 17 of which were informative. Of the three genes used, calmodulin gene had the most SNPs and indels (Table 4). Among the isolates that amplified for this gene, 1, 30 and 37, all from Eastern Kenya, were identified as *A. minisclerotigenis* in agreement with the results in section Identification and analysis of polymorphism in *A.flavus* b-tubulin gene above. The remaining isolates were 100% identical to *A. flavus* sequences in NBCI with exception of isolate 90, which was 100% identical to *A. parasiticus* sequences in the genebank. Phylogenetic tree-based on calmodulin gene data indicates 2 major clades, namely *A. parasiticus*—forming one clade while *A. flavus* and *A. minisclerotigenis* forming a second clade. Several sub-clades for *A. flavus* are observed indicating a wide variation among *A. flavus* species (Figure 4).

**Table 4:** *Aspergillus* sp. isolates showing nucleotide deletions and insertions on calmodulin gene.

**Nucleotide position**	**Base substitution**	**Base insertion**	**Isolates**	**Identity - NCBI database (100%)**	**Identical NCBI accession numbers**
32		T	1, 30, 37	*A. minisclerotigenes*	HM803026.1
			5, 14, 21, 34, 44, 107	*A. flavus*	KJ175547.1 LT558733.1
			90	*A. parasiticus*	KJ175554.1
32	Indel		39, 43, 60, 62, 64, 76, 108, 150	*A. flavus*	KX306820.1
82		T	1, 30, 37	*A. minisclerotigenes*	
			34, 39, 43, 44, 60, 62, 64, 76, 108, 150	*A. flavus*	
			90	*A. parasiticus*	
82	Indel		5, 14, 21, 107	*A. flavus*	KJ175559.1
95	T with A		5, 12, 21, 107	*A. flavus*	
106	C with T		5, 12, 21, 34, 107	*A. flavus*	
129	A with G		90	*A. parasiticus*	
129	T with G		90	*A. parasiticus*	
136	C with A		90	*A. parasiticus*	
212	T with C		1, 30, 37	*A. minisclerotigenes*	
238	C with T		90	*A. parasiticus*	
333	C with T		1, 30, 37	*A. minisclerotigenes*	
333	Indel		90	*A. parasiticus*	
356	C with T		90	*A. parasiticus*	
364		C	90	*A. parasiticus*	
366	T with C		90	*A. parasiticus*	
375	G with A		90	*A. parasiticus*	
381	G with T		1, 30, 37	*A. minisclerotigenes*	
			34	*A. flavus*	
			90	*A. parasiticus*	
414	C with T		5, 107	*A. flavus*	
492	T with C		1, 30, 37	*A. minisclerotigenes*	
			34	*A. flavus*	
			90	*A. parasiticus*	
531	C with T		34	*A. flavus*	

#### Identification and analysis of polymorphism in *A. flavus* ITS region

**Figure 5:**
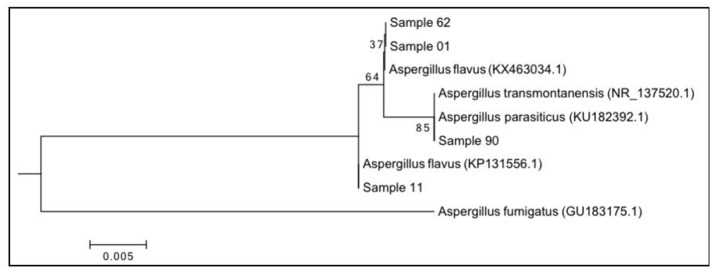
Molecular phylogenetic analysis of the ITS region by Maximum Likelihood method based on the Tamura 3-parameter model at 1000 bootstraps. The tree is drawn to scale, with branch lengths measured in the number of substitutions per site. Representative isolates are used. Evolutionary analyses were conducted in MEGA6.

Four hundred and sixty characters were used in the analysis of ITS gene sequences. The ITS region was conserved in most regions for these isolates. Five SNPs were observed (Table 5). All isolates were identified as *A. flavus* with identical similarity (100%), excepted strains 1 and 28 which possessed a heterozygous loci, and matched (99%) to *A. flavus*. Isolate 90 was 100% identical to *A*. *transmontanensis* and *A. parasiticus* sequences in the NCBI genebank. Phylogenetic analysis of the sequence data of the ITS region shows isolates 1 and 28 formed a clade with a closer relationship with *A. parasiticus* (Figure 5). Variation exists among *A. flavus* isolates; those with SNPs (1, 28, 90) are closer in relationship.

**Table 5:** *Aspergillus flavus* isolates showing nucleotide deletions and insertions on ITS region.

**Nucleotide position**	**Base substitution**	**Genotype**	**Identity to NCBI database (100%)**	**Identity to NCBI database (99%)**	**Identical NCBI numbers**
46	A with G	90	*A. transmontanensis*		NR137520.1
			*A. parasiticus*		KV182392.1
46	Indel	90	*A. parasiticus*		
50	A with A/G	1		*A. flavus*	KY859367.1
65	T with G	90			
66	Indel	90	*A. parasiticus*		
117	G with A	1, 62	*A. flavus*		
		90	*A. parasiticus*		
117	G with A/G	28		*A. flavus*	KY859367.1
Nil	Nil^*^	4, 5, 10, 11, 12, 14, 15, 21, 30, 34, 35, 37, 41, 42, 43, 44, 46, 47, 54, 55, 59, 60, 63, 64, 65, 69, 75, 76, 82, 83, 84, 85, 87, 88, 95, 96, 99, 106, 108, 109	*A. flavus*		KY859367.1

* *Nil means zero*.

#### Aflatoxin gene profile

The PCR results placed the isolates into four groups based on their patterns of amplification products: group I (28 strains) characterized by presence of all four amplicons; group II (2 strains) and III (3 strains), showing two (aflD, aflP) and one of the three (aflD, aflP, aflQ) amplicons, respectively; and group IV (51 strains) are characterized by a total absence of PCR products (Table 6). Strains belonging to all groups produced AFB1 and AFB2 on CYA, except for 63 and 97 in Group 1. CYA favored AF production compared with YESA. Only 5 (6%) isolates belonging to Group IV, gave consistent results for the absence of the four AF genes, and proved an inability to produce both AFB1 and AFB2 on YESA. Table 7 shows the isolates and aflatoxin production. Some *A.miniscleotigenes* isolates produced AFB's only while some *A. flavus* isolates produced both AFB's and AFG's inconsistent with the conventional chemotypes of the corresponding species.

**Table 6:** Aflatoxigenic gene profile of *Aspergillus flavus* isolates and aflatoxin production.

		**Gene prescence**				**AFB1**^a^ **production**		**AFB2**^a^ **production**	
**Group**	**Isolate**	**aflD (nor-1)**	**aflQ (ordA)**	**aflP (omtA)**	**aflR**	**AFB1 (CYA)**	**AFB1 (YESA)**	**AFB2 (CYA)**	**AFB2 (YESA)**
1	1, 4, 5, 14, 16, 28–34, 37-40, 42, 44, 47, 48, 51, 54, 58, 62–66, 84, 86, 87, 97	+	+	+	+	All except 63	+	All except 63, 97	All except 5, 16, 30 63, 86,
11	2, 90	+	−	+	−	+	+		Only 90
111	36, 67, 85, 107, 69			+		+	+		
	7, 76	+				+	+		
	71		+			+	+		
1V	3, 9, 10, 11, 12, 17–27, 35, 41, 43, 45, 46, 52, 53, 55−57, 59–61, 72–75, 78–83, 88, 89, 94–102, 106, 108, 109	−	−	−	−	+	All except 21, 41, 74, 108, 109		All except 9, 12, 17, 21, 35, 41, 53, 74, 81, 101, 108, 109

a *Fungal cultures were grown in CYA and YESAA medium for 7 days at 28°C*.

**Table 7:** *Aspergillus* isolates identity using three genes and aflatoxin production.

**Culture media**	**Aflatoxin produced**	**Isolate identity from NBCI blast**	**Grouping of isolate identity from NCBI database using various genes**			***N* total**
			**β-tubulin**	**Calmodulin**	**ITS**	
CYA	Both AFB's and AFG's	*A minisclerotigenes*	4, 10, 54, 67,			27
		*A. flavus*	2, 5, 8, 11, 12, 13, 14, 16, 17, 18, 26, 29, 36, 40, 46, 47, 58, 68, 72, 77, 93, 102, 105	2, 4, 5, 8, 10, 11, 12, 13, 14, 16, 17, 18, 26, 29, 36, 40, 46, 47, 54, 58, 67, 68, 72, 77, 93, 102, 105	2, 4, 5, 8, 10, 11, 12, 13, 14, 16, 17, 18, 26, 29, 36, 40, 46, 47, 54, 58, 67, 68, 72, 77, 93, 102, 105	
	AFB's	*A minisclerotigenes*	1, 30, 37, 42, 84	1, 30, 37		66
		*A. flavus*		3, 6, 7, 9, 15, 19, 20, 21, 25, 27, 28, 33, 34, 35, 38, 39, 41, 42, 44, 45, 49, 50, 52, 53, 55, 57, 59, 61, 64, 65, 66, 69, 70, 71, 73, 74, 75, 78, 80, 81, 82, 83, 84, 85, 86, 87, 89, 91, 92, 94, 95, 96, 97, 98, 99, 100, 101, 103, 104, 106, 107, 108	1, 3, 6, 7, 9, 15, 19, 20, 21, 25, 27, 28, 30, 33, 34, 35, 37, 38, 39, 41, 42, 44, 45, 49, 50, 52, 53, 55, 57, 59, 61, 64, 65, 66, 69, 70, 71, 73, 74, 75, 78, 80, 81, 82, 83, 84, 85, 86, 87, 89, 90, 91, 92, 94, 95, 96, 97, 98, 99, 100, 101, 103, 104, 106, 107, 108	
		*A. parasiticus*	90	90		
		Unnamed. Only 99% match of *A. flavus* in NCBI database			1, 28	
	No aflatoxin	*A. flavus*	63	63	63	1
YESA	Both AFB's and AFG's	*A minisclerotigenes*	1	1		6
		*A. flavus*	11, 82, 93, 102	11, 82, 93, 102	11, 82, 93, 102	
		*A. parasiticus*	90	90,	90	
		Unnamed. Only 99% match of *A. flavus* in NCBI database			1	
	AFB's	*A minisclerotigenes*	4, 10, 30, 37, 42, 54, 67, 84	30, 37		85
		*A. flavus*	2, 3, 5, 6, 7, 8, 9, 12, 13, 14, 15, 16, 17, 18, 19, 20, 25, 26, 27, 28, 29, 33, 34, 35, 36, 38, 39, 40, 44, 45, 47, 48, 49, 50, 52, 53, 55, 57, 58, 59, 61, 63, 64, 65, 66, 68, 69, 70, 71, 72, 73, 75, 76, 77, 78, 80, 81, 83, 85, 86, 87, 89, 91, 92, 94, 95, 96, 97, 98, 99, 100, 101, 103, 104, 105, 106, 107	2, 3, 4, 5, 6, 7, 8, 9, 10, 12, 13, 14, 15, 16, 17, 18, 19, 20, 25, 26, 27, 28, 29, 33, 34, 35, 36, 38, 39, 40, 42, 44, 45, 47, 48, 49, 50, 52, 53, 54, 55, 57, 58, 59, 61, 63, 64, 65, 66, 67, 68, 69, 70, 71, 72, 73, 75, 76, 77, 78, 80, 81, 83, 84, 85, 86, 87, 89, 91, 92, 94, 95, 96, 97, 98, 99, 100, 101, 103, 104, 105, 106, 107	2, 3, 4, 5, 6, 7, 8, 9, 10, 12, 13, 14, 15, 16, 17, 18, 19, 20, 25, 26, 27, 29, 30, 33, 34, 35, 36, 37, 38, 39, 40, 42, 44, 45, 47, 48, 49, 50, 52, 53, 54, 55, 57, 58, 59, 61, 63, 64, 65, 66, 67, 68, 69, 70, 71, 72, 73, 75, 76, 77, 78, 80, 81, 83, 84, 85, 86, 87 89, 91, 92, 94, 95, 96, 97, 98, 99, 100, 101, 103, 104, 105, 106, 107	
		Unnamed. Only 99% match of *A. flavus* in NCBI database			28	
	No aflatoxins	*A. flavus*	21, 41, 74, 108, 109	21, 41, 74, 108, 109	21, 41, 74, 108, 109	5

## Discussion

Use of molecular markers in this study separates a population initially named as *A. flavus* using morphological and cultural (AFPA) characteristics into two species, namely *A. flavus, A. minisclerotigenes. A. minisclerotigenes* was common in Eastern Kenya while only one isolate of the species was identified from the Rift Valley using the β-tubulin gene marker. *A. flavus* was common in both regions. The single isolate that was close in identity to *A. parasiticus* was from the Rift Valley. Further, the NJ tree based on β-tubulin gene sequences indicates that *A. flavus* with *A. parasiticus* are closely related forming a clade distinct from *A. minisclerotigenes*. Nyongesa et al. (2015), reported that *A. parasiticus* was frequently isolated from Rift Valley compared to Eastern Kenya. These results show spatial distribution between *A. flavus* and *A. minisclerotigenes* and could suggest different evolutionary origins of the two species. Chang et al. (2009) observed that *A. flavus* descended from the ancestor of *A. parasiticus*, while *A. minisclerotigenes* descended from a different ancestor. Calmodulin gene sequences showed a wider variation among the *A. flavus* isolates separating few as *A. minisclerotigenes* compared with β-tubulin gene sequences, and none for ITS gene sequences. Analysis of the ITS region also showed variation among the *A. flavus* isolates.

The mycotoxin metabolic profile of the *Aspergillus* strains was highly variable suggesting possible high levels of genetic recombination among members of this species (Taylor et al., 1999). For example, isolate 90, which was identified *as A. parasiticus* produced CPA, which is not a known characteristic for this species. The same applied to some isolates of *A. minisclerotigenes* producing only AFB's, while some isolates of *A. flavus* produced both AFB's and AFG's. *A. minisclerotigenes* is known to produce both AFB's and AFG's while some isoltes of *A. flavus* have been classified as producing only AFBs (Pildain et al., 2008) while others produce both (Okoth et al., 2012). Similarly, Olarte et al. (2012) demonstrated high AF heritability, genetic variability and recombination in the AF gene cluster, analogous to spontaneous recombination during sexual reproduction in natural populations.

Knowledge of relative stability of characters among isolates of this species is important in the selection and use of non-toxigenic strains for the biological control of toxigenic ones. Further, *A. flavus* is an important taxa in food safety and as human pathogen. However, information of the relative phylogenetic distances at which these characters are acquired and lost, is yet to be established.

It was not always possible to distinguish aflatoxigenic species from non-aflatoxigenic species using PCR detection of AF biosynthesis genes, as some of the results were conflicting with the mycotoxin metabolite analysis. Both Gallo et al. (2012) and Levin (2012) reported similar observation and attributed this to inter- and intra-specific genetic mutations within the primers' targeted binding site. Fakruddin et al. (2015) tested 15 isolates of *A. flavus* for aflatoxigenic genes (aflD, *aflM, aflP, aflQ, aflS, aflO*, and *aflR*), and only one isolate possessed all of the 7 genes tested. The remaining isolates varied widely in terms of which genes among the 7 they possessed. The authors explained this to the inability of the primers used to amplify the genes. A few studies have reported a correlation between AF gene expression and AF production using RT-PCR and real time PCR targeting aflD, aflQ, aflO, aflP, aflR, and aflS genes (Sweeney et al., 2000; Jamali et al., 2013; Davari et al., 2015; Fakruddin et al., 2015; Baquiao et al., 2016). Mahmoud (2015) reported a correlation between PCR amplification of four aflatoxin biosynthetic pathway genes (*aflD, aflM, aflP*, and *aflQ*) with the AF production capability of *A. flavus* isolates from stored peanuts in Egypt. The same authors, however, noted that the expression of *aflD* and *aflQ* was a good marker for differentiating toxigenic from atoxigenic isolates. Accinelli et al. (2008) and Lourenco et al. (2007) did not find any correlation between profiles of aflatoxigenic *A. flavus* isolates and AFB1 concentrations in the soil. Jamali et al. (2013) recorded a correlation with AF produced. This study confirms the lack of correlation between the presence of the *aflD, aflQ, aflP, aflR* genes & AFB1 and AFB2 production, and promotes the usefulness of using both molecular and mycotoxin metabolite profiles in identification of the isolates. Though microbial and immunological methods are considered costly, time-consuming and may produce false-positives and/or false-negative results, a combination of these techniques together with molecular methods yields reliable results. Molecular biology methods combined with morphology and physiology reveal the diversity within the *A. flavus* species, and the need for further studies to enable identification of varieties. To date, the only clear morphological variation is the size of sclerotia. Small sclerotial *A. flavus* and *A. minisclerotigenes* are known to produce large amounts of aflatoxins, and these are most frequent in aflatoxin-hot spot Eastern region of Kenya.

## Author contributions

SO: Conceived and designed the experiments; SO, MD, AV, JD, SL, SD, MK, and JH: Generated data; JN, MD, and SO: Analyzed data; MD, SO, and SD: Wrote the manuscript.

### Conflict of interest statement

The authors declare that the research was conducted in the absence of any commercial or financial relationships that could be construed as a potential conflict of interest.
