# Supplementary material for: Genetic and Toxigenic Variability within Aspergillus flavus Population Isolated from Maize in Two Diverse Environments in Kenya
Source: Front Microbiol. 2018 Jan 26;9:57. doi: 10.3389/fmicb.2018.00057 (PMC5790802; doi:10.3389/fmicb.2018.00057)
Supplement: Supplementary file 1 [file Table1.docx]

**Supplementary Table 1. NCBI^1^ Accession Numbers for ITS^2^ gene sequences for *Aspergillus flavus* isolates from maize kernels**

| Isolate Code | Origin | Identity - NCBI database | NCBI  accession numbers |
| --- | --- | --- | --- |
| 001 | Kenya | *A. flavus* | MG596351 |
| 011 | Kenya | *A. flavus* | MG596352 |
| 028 | Kenya | *A. flavus* | MG596353 |
| 062 | Kenya | *A. flavus* | MG596354 |
| 090 | Kenya | *A. parasiticus* | MG596355 |
| 109 | Kenya | *A. flavus* | MG596356 |

^1^NCBI = National Centre for Biotechnology Information

^2^ITS = Internal transcribed spacer region

**Supplementary Table 2. NCBI^1^ Accession Numbers for Bt^2^ gene sequences for *Aspergillus flavus* isolates from maize kernels**

| Isolate Code | Origin | Identity - NCBI database | NCBI  accession numbers |
| --- | --- | --- | --- |
| 001 | Kenya | *A. minisclerotigenes* | MG573337 |
| 042 | Kenya | *A. minisclerotigenes* | MG573338 |
| 004 | Kenya | *A. minisclerotigenes* | MG573339 |
| 028 | Kenya | *A. flavus* | MG573340 |
| 067 | Kenya | *A. minisclerotigenes* | MG573341 |
| 088 | Kenya | *A. flavus* | MG573342 |
| 090 | Kenya | *A. parasiticus* | MG573343 |

^1^NCBI = National Centre for Biotechnology Information

^2^Bt = Beta – tubulin gene

**Supplementary Table 3. NCBI^1^ Accession Numbers for CaM^2^ gene sequences for *Aspergillus flavus* isolates from maize kernels**

| Isolate Code | Origin | Identity - NCBI database | NCBI  accession numbers |
| --- | --- | --- | --- |
| 001 | Kenya | *A. minisclerotigenes* | G573344 |
| 005 | Kenya | *A. flavus* | G573345 |
| 014 | Kenya | *A. flavus* | G573346 |
| 030 | Kenya | *A. flavus* | G573347 |
| 034 | Kenya | *A. minisclerotigenes* | G573348 |
| 037 |  | *A. minisclerotigenes* | G573349 |
| 060 |  | *A. flavus* | G573350 |
| 107 |  | *A. flavus* | G573351 |
| 021 |  | *A.flavus* | G573352 |
| 076 |  | *A. flavus* | G573353 |
| 090 | Kenya | *A. parasiticus* | G573354 |

^1^NCBI = National Centre for Biotechnology Information

^2^CaM= Calmodulin gene
